# On the learning of addictive behavior: Sensation-seeking propensity predicts dopamine turnover in dorsal striatum

**DOI:** 10.1007/s11682-021-00509-5

**Published:** 2021-08-21

**Authors:** Natalie Hong Siu Chang, Yoshitaka Kumakura, Arne Møller, Jakob Linnet, Dirk Bender, Doris J. Doudet, Manouchehr Seyedi Vafaee, Albert Gjedde

**Affiliations:** 1 Department of Clinical Research, University of Southern Denmark, Odense C, DK-5000 Denmark; 2 Department of Diagnostic Radiology and Nuclear Medicine, Saitama Medical University, 1981 Kamoda, Kawagoe, Saitama 350-8550 Japan; 3 Department of Nuclear Medicine and PET Centre, Aarhus University Hospital, DK-8200 Aarhus N, Denmark; 4 Gambling Disorder & BED Clinic, Department of Occupational and Environmental Medicine, Odense University Hospital, Odense C, Dk-5000 Denmark; 5 Department of Medicine, Division of Neurology, University of British Columbia, Vancouver, B.C. V6T 2B5 Canada; 6 Department of Clinical Research, BRIDGE, University of Southern Denmark, Odense M, DK-5230 Denmark; 7 Department of Neuroscience, University of Copenhagen, Copenhagen, DK-2200 Denmark; 8 Department of Clinical Medicine, University of Southern Denmark, Odense C, DK-5000 Denmark; 9 Translational Neuropsychiatry Unit, Aarhus University, Aarhus, DK-8000 Denmark

**Keywords:** Neuroscience, Brain imaging, Sensation-seeking, Dopamine transmission

## Abstract

We asked if sensation-seeking is linked to premorbid personality characteristics in patients with addictive disorders, or the characteristics follow the sensation-seeking activity. We interpreted the former as a state associated with normal rates of dopamine synthesis, and the latter as a trait of individuals with abnormally high rates of synthesis. We previously determined dopaminergic receptor density in striatum, and we now tested the hypothesis that an elevated dopaminergic condition with increased extracellular dopamine and receptor density follows increased dopamine synthesis capacity in highly sensation-seeking individuals, as measured by positron emission tomography of 18 men with tracer fluorodopa (FDOPA). We detected a site in left caudate nucleus where the volume of distribution of FDOPA-derived metabolites correlated negatively with FDOPA metabolite turnover, consistent with decreased metabolite breakdown in highly sensation-seeking subjects. High rates of sensation-seeking attenuated the dopamine turnover in association with a low rate of dopamine recycling, low dopamine oxidation, and elevated extracellular dopamine and receptors in caudate nucleus. In contrast, low rates of sensation-seeking were associated with rapid dopamine recycling, rapid dopamine oxidation, low extracellular dopamine, and low receptor density. We conclude that the modulation of dopaminergic neurotransmission associated with sensation-seeking is a state of sensation-seeking, rather than a trait of personality following abnormal regulation of dopaminergic neurotransmission.

**Supplementary Information:**

The online version contains supplementary material available at 10.1007/s11682-021-00509-5.

## Introduction

In the brain, neurotransmission by dopamine (DA) plays a key role in behavior associated with motivation. Dopaminergic neurotransmission is implicated in reward, motivation, decision making, reinforcement learning, and behavioral addiction, which may compel individuals to engage in risky activities. Sensation seeking is associated with risky behavior, including pathological gambling, alcohol and drug use in adolescence, and predicts development of substance abuse (Zuckerman, 1994). We previously documented increases of dopamine receptor density and extracellular dopamine in highly sensation-seeking individuals (Gjedde et al., 2010). Highly sensation seeking individuals are more sensitive and vulnerable than less sensation seeking individuals to the reinforcing effects of d-amphetamine, (Kelly et al., 2006; Stoops et al., 2007) and highly sensation seeking rats tended to display a much higher propensity to self-administer psychostimulants (Blanchard et al., 2009). Also, human risk-taking can be directly manipulated by the selective D_2_/D_3_ agonist cabergoline, but the effect depends on the level of baseline sensation-seeking (Norbury et al., 2013).

Although results of investigations into positive subjective responses to d-amphetamine are not entirely consistent, numerous studies lend support to the claim of increased degrees of substance abuse among highly sensation-seeking individuals (White et al., 2006; White & White, 2006). In contrast, it is possible that high sensation seeking is a form of learned behavioral addiction, imposed by the initial engagement in sensation-seeking activity. Thus, the assessment of causality is confounded by effects of the additional use of psychostimulants, and the role of learning in the vulnerability to substance abuse remains a matter of debate.

The high prevalence of comorbid substance abuse in patients with schizophrenia is a matter of debate too. Among hypotheses from different studies (Chambers et al., 2001; Dixon, 1999; Regier et al., 1990), the addiction vulnerability explanation suggests that addiction and schizophrenia are primary disease symptoms with common abnormalities, where positive reinforcing effects of drug reward and reduced inhibitory control over drug-seeking behavior are facilitated (Chambers et al., 2001). With the classic observation that the psychoticism trait of Eysenck (Eysenck & Zuckerman, 1978) was correlated positively with sensation-seeking, it is possible to invoke a continuum of mental states, personality traits, and psychiatric symptoms, where sensation seeking activity, and contact with reality, may interchange with periods of loss of contact with reality (Guillem et al., 2005). The continuum of the reality awareness dimensions can be extended to the general population as a whole with variable sensation seeking levels (Van Os, 2003).

Previous reports from this group showed that DA synthesis capacity is elevated in patients with the trait of psychosis (Reith et al., 1994) or schizophrenia (Howes et al., 2012; Kumakura et al., 2007). The purpose of the present study was to test whether abnormalities of presynaptic dopamine turnover are as likely to be causes as consequences of sensation-seeking, when measured with fluorine-18-labeled FDOPA and PET. To do so, we employed a novel method of analysis of DA synthesis capacity (Kumakura et al., 2007; Kumakura et al., 2006). Previous FDOPA PET studies had methodological limitations, leaving sources and processes of aberrant dopamine metabolism unaccounted for. By means of specific pharmacokinetic analysis of FDOPA metabolism in brain (Kumakura et al., 2007; Kumakura et al., 2006), we tested the hypothesis that DA synthesis capacity of human striatum would correlate with severity of sensation-seeking propensity, as follow-up to the previously documented increases of dopamine receptor density and extracellular dopamine in highly sensation-seeking individuals (Gjedde et al., 2010). The application of FDOPA brain PET imaging with arterial blood sampling yielded three separate kinetic indices, including the coefficient of transfer (clearance) of FDOPA from the circulation to the brain (*K*), the rate of loss of DA metabolites from brain tissue (*k*_loss_), and the apparent volume of distribution of fluorodopamine (*V*_D_), indicative of dopamine storage capacity and turnover.

After ethics committee permission, we studied 18 individuals recruited after advertisement in public media and at a treatment center. We continued to advertise until a sufficient distribution of consecutive individual sensation-seeking scores had been obtained. We decided that the final distribution was sufficient to match the distribution included in the previous report by this group of dopamine binding in relation to sensation-seeking (Gjedde et al., 2010).

## Method

The methods closely followed the approach to the previously documented increases of dopamine receptor density and extracellular dopamine in more or less highly sensation-seeking individuals (Gjedde et al., 2010).

### Study Design

We recruited participants by local advertisement in newspapers and at local treatment centers, and scored the participants in the range of the 40-point Zuckerman sensation-seeking scale (ZSSS).

We did not preselect participants on the basis of score. The ranking is based on the response to questions about individual proclivity to engage in novel or risky activities. At first contact, we accepted only healthy, right-handed candidates who took no medication for central nervous disorders and had no metallic foreign objects. We screened the candidates for signs of psychological or psychiatric symptoms by means of a formal Structured Clinical Interview for the DSM-IV (SCID).

Twenty-one men aged 27.6 ± 6.5 (mean ± SD, range 20-38) passed the first contact and then gave written informed consent to the study as approved by the official Central Danish Regional Science Ethics Committee. All research was performed in accordance with relevant guidelines/regulations. Of these, only 18 men completed the experiments for technical reasons. The mean age of the 18 successful participants was 27.7 ± 6.4 (mean ± SD, range 20-38), and the mean of their ZSSS scores was 24.4 ± 3.8 (mean ± SD, range 15-30) with a median of 25 used to divide the participants into two groups, below and above ZSSS of 25. The ZSSS scores of the two groups averaged 21.6 ± 3.0 (SD) for the low group (n = 9) and 27.3 ± 1.7 (SD) for the high group (n = 9).

### PET sessions and plasma sampling procedures

Subjects fasted overnight before the PET experiment, and a dose of 200 mg (p.o.) Carbidopa (Merck Sharpe and Dohme) was given one hour before the PET recording to minimize the decarboxylation of FDOPA in peripheral tissues (Cumming et al., 1993). Subjects reclined on the scanning bed of the ECAT High Resolution Research Tomograph (HRRT), a dedicated human brain PET (CTI/Siemans, Knoxville, TN), with their heads comfortably immobilized using a custom-made head-holder. After a brief attenuation scan, we started dynamic 3D emission recording of 180 minutes (including a 30 min. intermission, 120-150 min.) upon intravenous injection of FDOPA (200 MBq). FDOPA was produced by electrophilic fluorination, applying an adopted standard method (Namavari et al., 1992). We collected 27 arterial blood samples at intervals increasing from 20 seconds to 30 minutes, and measured the total radioactivity from fluorine-18 in plasma in a well-counter cross-calibrated to the tomograph. We also measured the fractions of untransformed FDOPA and its major plasma metabolite, 3-O-methyl-FDOPA (OMFD) in selected plasma samples (2.5, 5, 15, 25, 35, 60, 90, 120, 150, and 180 minutes) by reverse-phase high performance liquid chromatography (Melega et al., 1991). We then calculated plasma input functions for FDOPA and OMFD by fitting bi-exponential functions to the measured fractions (Gillings et al., 2001).

### PET image analysis

Representative transaxial brain maps of the magnitude of quadratic coefficient were calculated voxelwise without correction for multiple comparisons by least-squares optimization of the linear relationship, where the Zuckermann sensation-seeking score is the explanatory variable and the parameters of the inlet-outlet model of each voxel are the dependent variables with superimposition on the standard MRI brain atlas of the MNI for precise anatomical identification. We decided that the prior hypothetical selection of regions-of-interest rendered multiple comparisons unnecessary.

We realigned the whole dynamic frame sequences for head motion correction, and registered the PET images to the MNI stereotaxic brain using the high resolution MR images and the mutual information maximization algorithm (Collignon et al., 1995). We corrected each dynamic frame for the radioactivity contribution from the brain-penetrating FDOPA metabolite, OMFD. To this end, we applied a modified one-tissue compartment model to the time-activity curves (TACs) of cerebellum (48.3 cm^3^), and calculated the blood-brain clearance (*K*_1_, ml cm^− 3^ min^− 1^), the diffusion rate from brain (*k*_2_, min^− 1^), and their ratio (*V*_*e*_, ml cm^− 3^) for both FDOPA and OMFD. This model entails the constraints of a common blood-brain partition ratio for the two large neutral amino acids (FDOPA: $K_{\mathrm {1}}^{\mathrm {D}}$/$K_{\mathrm {2}}^{\mathrm {D}}$, OMFD: $K_{\mathrm {1}}^{\mathrm {M}}$/$K_{\mathrm {2}}^{\mathrm {M}}$, ml cm^− 3^). It also assumes that the ratio of the two unidirectional clearances ($K_{\mathrm {1}}^{\mathrm {M}}$/$K_{\mathrm {1}}^{\mathrm {D}}$) should be constant (*q*; fixed at 1.5), (Cumming & Gjedde, 1998; Gjedde et al., 1991) based on a systematic study of the effect of *q* on the calculation of FDOPA kinetics (Léger et al., 1998). We then recovered the global brain OMFD TAC for all the frames, and subtracted the OMFD radioactivity at each frame from the appropriate dynamic emission frame, as described in detail elsewhere (Kumakura et al., 2005).

We used the pharmacokinetic inlet-outlet model (IOM), (Kumakura et al., 2006) which accommodates the intrinsic blood-brain clearance of FDOPA corrected for elimination of the decarboxylated metabolites (*K*, ml cm^− 3^ min^− 1^), and a first-order rate constant, expressing the diffusion from brain of [^18^F]fluorodopamine together with its acidic metabolites from brain as a single compartment (*k*_loss_, min^− 1^). Of necessity, and by definition, the term *k*_loss_ is a kinetic simplification based upon the assumption that an equilibrium is obtained between the sum of all [^18^F]fluorodopamine pools in the vesicles and cytosol of dopamine neurons, and other cells containing DOPA decarboxylase, and the several pools of deaminated metabolites, irrespective of their site of formation (Cumming et al., 1997; Deep et al., 1997; Deep et al., 1997).

The magnitude of the ratio *K*/*k*_loss_ is a quantitative measure of the effective distribution volume (*V*_ED_) of decarboxylated FDOPA metabolites trapped in brain (ml cm^− 3^). The measure of brain radioactivity includes activity distributed in the plasma volume (*V*_0_, ml cm^− 3^), as well in the precursor pool (*V*_*f*_, ml cm^− 3^) and the effective distribution volume. The composite of the three distribution volumes (*V*_0_, *V*_*f*_, and *V*_ED_) constitutes the total tracer distribution (*V*_D_, ml cm^− 3^) as an index of the steady-state dopamine storage used in the present study. The IOM consists of a set of first-order differential equations that rearrange to a linearized solution, which permits us to calculate parametric maps of *V*_D_ (Kumakura et al., 2006). We used the linear regression with the spatial constraint (LRSC) method (Zhou et al., 2003) to reduce the noise-related bias in the voxel-wise calculation of *V*_D_.

### Statistical analysis

We first stratified the 18 subjects into two subgroups at the median of the ZSSS scores for preliminary subgroup comparison, and calculated mean *V*_D_ maps from the radioactivity distribution. Then, we calculated Δ*V*_D_ subtraction map and the associated voxel-wise unpaired t-values, to identify coordinates with the greatest t-value in striatum of the MNI standard brain. To test the hypothesis, we carried out voxel-wise Pearson’s correlation analysis to find a cluster of voxels where *V*_D_ maps were significantly correlated with the individual ZSSS scores. Using the cluster as the anatomical substrate, we obtained kinetic values of *V*_D_, *K*, and *k*_loss_ from the regression to the 18 subjects, and then tested each kinetic parameter with the individual ZSSS scores for linear correlation. We tested the non-linear hyperbolic relationship between *V*_D_ and *k*_loss_ with a one-phase exponential decay function. We determined the significance (P < 0.0001) from the magnitude of *R*^2^ value at the degrees of freedom (n = 15) of this non-linear regression analysis.

## Results

**Fig. 1:**
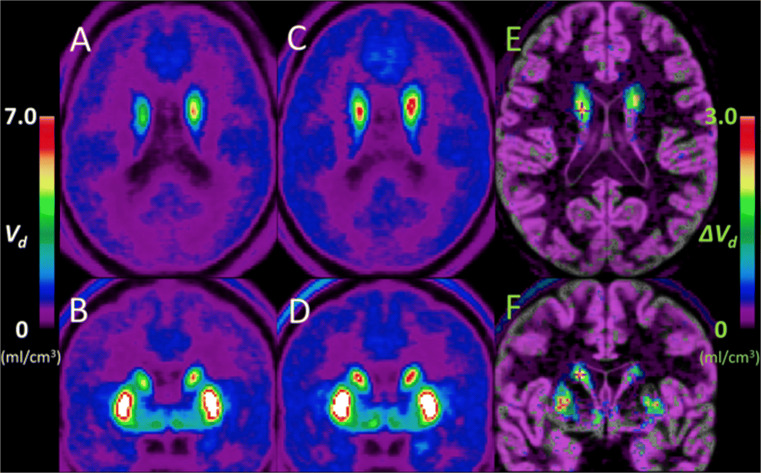
Mean *V*_D_ parametric maps of FDOPA for two stratified subgroups, together with subtraction maps. Mean images of nine subjects with ZSSS scores less than or equal to 25 (**A, B**), and those of the remaining nine male subjects with ZSSS scores greater than 25 (**C, D**) are shown on axial (**A, C**) and coronal image sections (**B, D**) in the common stereotaxic space. Here, the subtraction images (**E, F**) are superimposed on the gray matter MR images, and the crosshairs indicate the coordinates of the greatest Δ*V*_D_, and voxel wise t-value > 3.0 for the preliminary group comparison. Other areas (unmarked) of the subtraction planes did not reach statistical significance

**Fig. 2:**
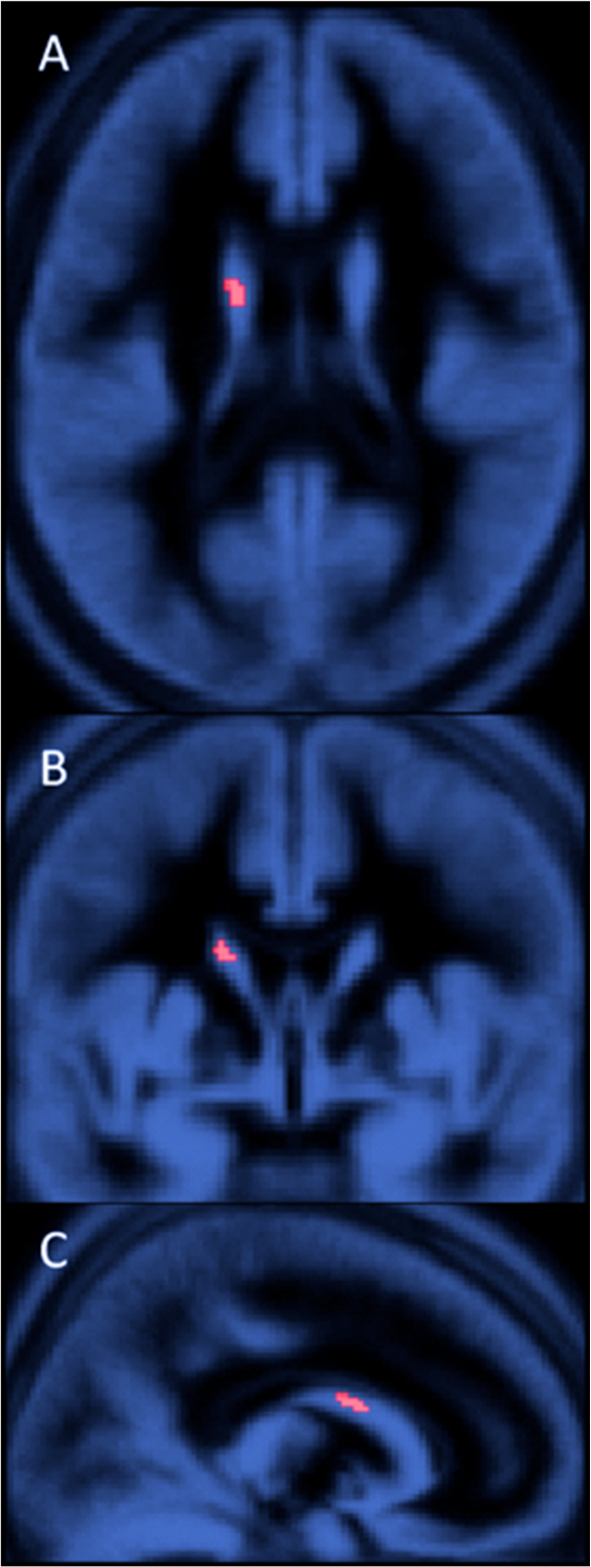
The cluster of voxels with t > 2.8 (red area) revealed by calculating the t-statistic map for voxel-wise linear regression between the individual FDOPA-*V*_D_ maps and scores of the Zuckerman sensation-seeking scale (N = 18). The axial (**A**), coronal (**B**), and sagittal (**C**) views are superimposed on the gray matter MRI image of the MNI standard brain. The red cluster of the left dorsal striatum includes the coordinates of (-15, 0, 20). The volume of the cluster is 145 mm^3^, occupying 80 voxels of the reconstructed images of the HRRT PET device

**Fig. 3:**
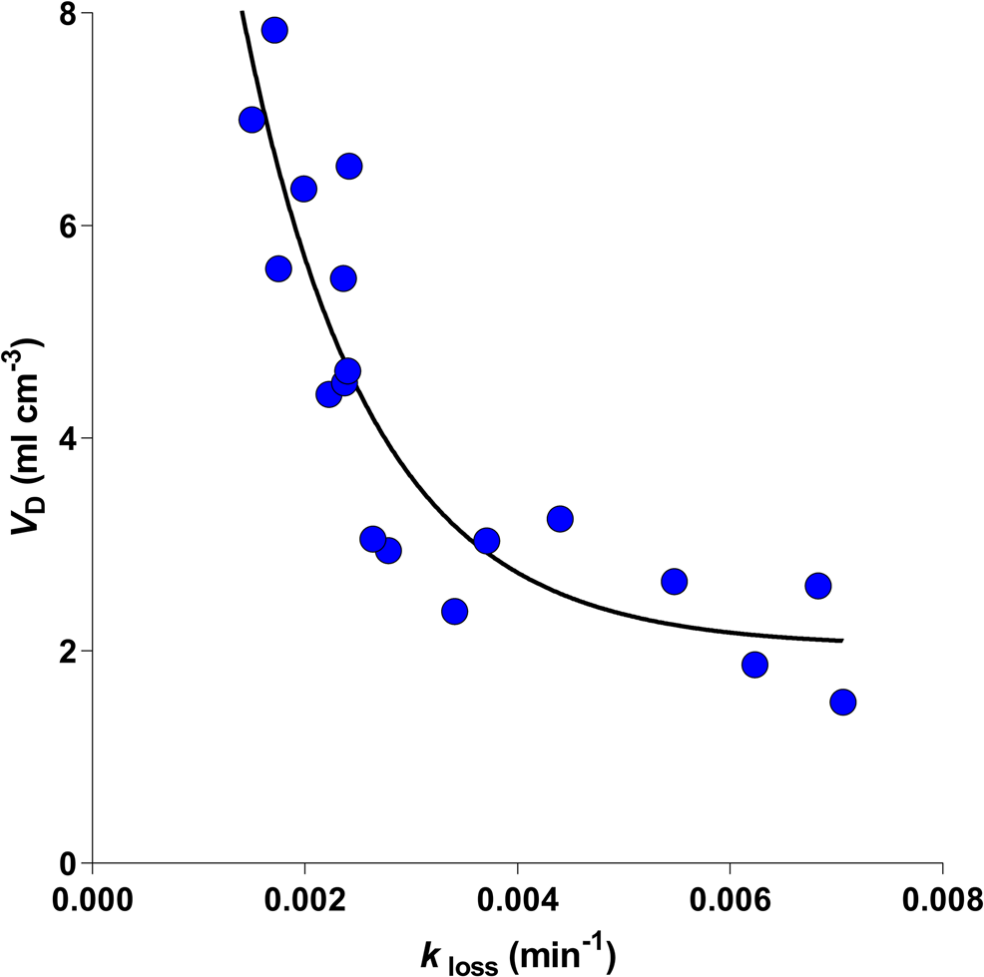
The significant hyperbolic relation between the apparent volume of distribution and turnover rate, calculated for the left dorsal striatum cluster in the group of 18 young healthy volunteers (*R*^2^ = 0.76, P < 0.0001). The hyperbolic relation between *V*_D_ and *k*_loss_ confirms no significant contribution of *K*, to the sensation-seeking propensity, as shown in Fig. 4b

**Fig. 4:**
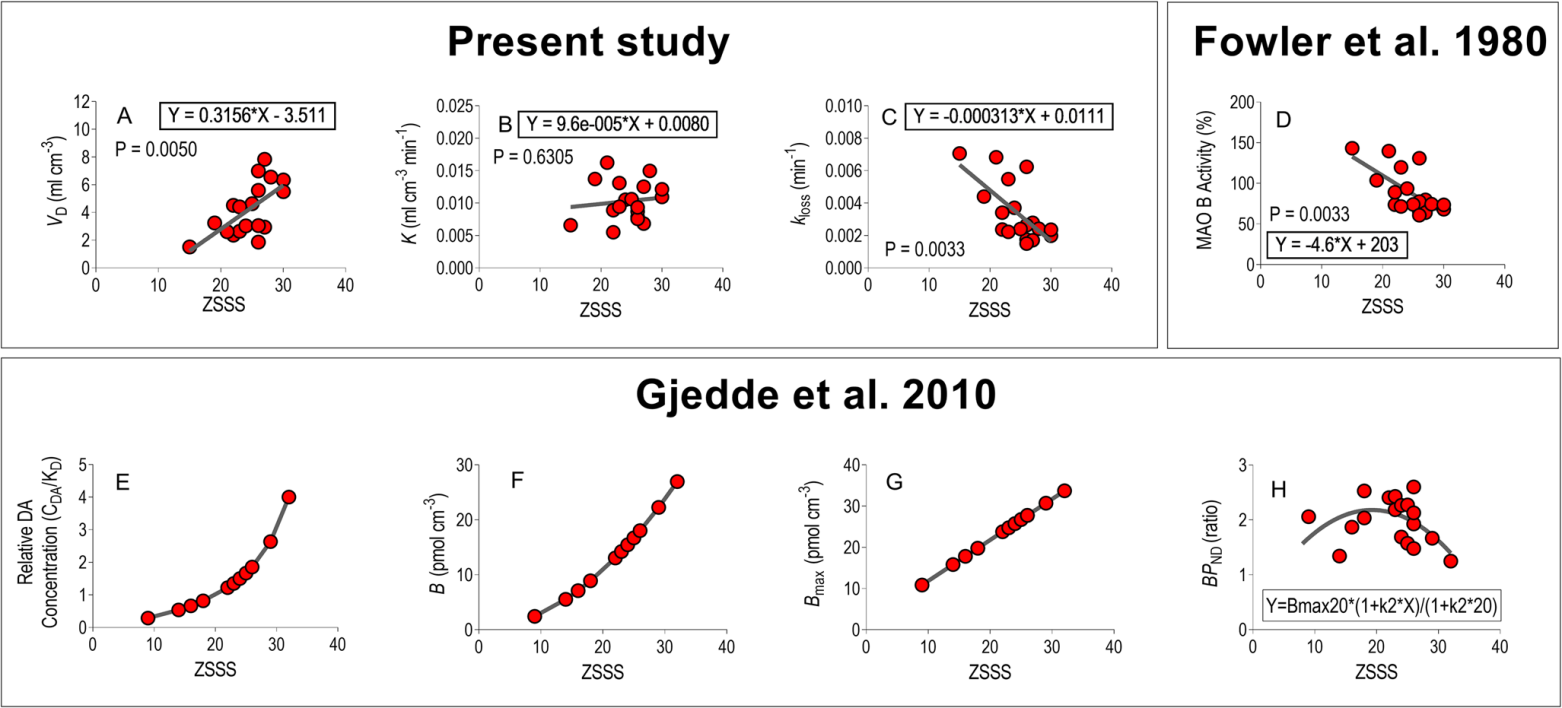
State or trait distributions of dopaminergic variables within populations of young healthy volunteers. Upper Row: Relationships between major variables of dopamine synthesis and dopaminergic neurotransmission as functions of sensation-seeking propensity, based on estimates obtained in the present study (left hand graphs) and for monoamine oxidase by Fowler et al., 1980 (right hand graph). Abscissae: Zuckerman Sensation-Seeking Score (ZSSS). Ordinates: Apparent distribution volumes of FDOPA (**A**: ml cm^− 3^); transfer coefficient of FDOPA (**B**: ml cm^− 3^ min^− 1^); rate of loss of dopamine metabolites from the brain (**C**: min^− 1^); relative MAO-B activity calculated from individual ZSSS scores (**D**: %, calculated from data of Fowler et al., 1980). The linear regression analyses for correlation between the ZSSS scores and FDOPA kinetic estimates of *V*_D_ (**A**) and *k*_loss_ (**C**), were based on the left dorsal striatum cluster Lower Row: Relationships between major variables of dopamine synthesis and dopaminergic neurotransmission as functions of sensation-seeking propensity, obtained by Gjedde et al., 2010 where the panels (**E-H**) show relative DA concentration (**E**: ratio); bound endogenous ligand (**F**: pmol cm^− 3^); maximum binding capacity (**G**: pmol cm^− 3^); and dopamine D2/D3 receptor availability (**H**: ratio).

First, maps of FDOPA-derived volumes of DA storage (*V*_D_), divided into the highest and lowest ZSSS value groups, were averaged in common space and subtracted to reveal a difference map of values at t > 3 (Fig. 1). Second, voxel-wise linear regression of the *V*_D_ maps against ZSSS for the entire population of 18 subjects were obtained as shown in Fig. 2 for t > 2.8. The individual values are shown in Fig. 3 for *V*_D_ vs. *k*_loss_ and as function of ZSSS in Fig. 4a.

### Parametric mapping

We used the inlet-outlet model (IOM) set of first-order differential equations that rearrange to a linearized solution that yielded parametric maps of *V*_D_ (Kumakura et al., 2006). The mean FDOPA *V*_D_ parametric maps of two subgroups of subjects, assigned as either below or above the median of the Zuckerman sensation-seeking scale (ZSSS) scores in the present population, are shown in Fig. 1, with the subtraction map indicating a significant difference in left dorsal striatum, where the peak t-value was greater than 3.0 (at coordinates x, y, z: 15mm, 0mm, 20mm). There was no significant difference in the mean *V*_D_ value of ventral striatum between the two subgroups, nor in cerebellum (Supplementary Figure 6).

To test the prediction of a correlation of *V*_D_ measures with scores of the ZSSS, we performed regression analysis on the former against the latter for the 18 healthy male volunteers included in the study. We detected voxels (145 mm^3^) with Student’s t-statistics greater than 2.8 by voxel-wise mapping of linear regression analysis between the *V*_D_-parametric maps and the ZSSS scores. To obtain average estimates, we defined a region representing the cluster of t values above the selected threshold (t > 2.8) shown in Fig. 2. The coordinates of the center of mass of this cluster are (x,y,z) 15 mm, 0 mm, 20 mm. For this cluster, the overall mean estimates of the three kinetic parameters *V*_D_, *K*, and *k*_loss_ were 4.2 ml cm^− 3^, 0.010 ml cm^− 3^ min^− 1^, and 0.0034 min^− 1^, respectively. The apparent volume of distribution (*V*_D_) revealed an inverse relation with the elimination and turnover constant (*k*_loss_), in the shape of the hyperbola shown in Fig. 3 (*R*^2^ = 0.76, P < 0.0001), as expected when the inherent clearance of FDOPA from plasma to brain (*K*) is unrelated to the sensation-seeking propensity.

### Linear regression analyses

We determined the linear correlations between the estimates of the remaining parameters of the region of interest and the individual ZSSS scores, as shown in Figs. 4**A-C**.The average individual regional estimates of the turnover rate constants *k*_loss_ were negatively correlated with the ZSSS scores (P = 0.0033) as shown in Fig. 4c, while the average regional estimates of net clearance from the circulation (*K*) were not correlated to the ZSSS scores (P = 0.63) as shown in Fig. 4b. It follows that the volume of dopamine storage *V*_D_ with the ZSSS scores must be highly positive, as shown in Fig. 4a.

## Discussion

We tested the association between the sensation-seeking propensity assessed by Zuckerman’s sensation-seeking scale, and states of dopamine synthesis and breakdown obtained by kinetic analysis of brain PET images after administration of FDOPA (Kumakura et al., 2007; Kumakura et al., 2006; Kumakura et al., 2005).

We reasoned that an elevation of DA synthesis capacity would correlate with the onset of higher-than-average sensation-seeking propensity if the intense sensation-seeking is an unusual but naturally occurring trait of the brain (null hypothesis). Alternatively, if no underlying abnormality of dopamine synthesis capacity can be detected, it follows that the sensation-seeking propensity can be regarded a state of mind that underlies the learning of an addictive behavior.

The test of the hypothesis revealed a site in the dorsal part of the left caudate nucleus where the dopamine storage capacity (*V*_D_) was inversely proportional to the dopamine turnover rate as indicated by the rate of loss (*k*_loss_), while the estimates of synthesis capacity (*K*) revealed no correlation with the scores of sensation-seeking propensity. The three parameters identified by the analysis define the kinetics of regional dopamine turnover and storage capacity, of which the DA synthesis capacity is known to be abnormal in patients with neuropsychiatric disorders, including Parkinson’s disease, schizophrenia, and alcoholism (Gjedde et al., 1993; Kumakura & Cumming, 2009; Kumakura et al., 2013). In normal control subjects, the typical kinetic estimates include low values of *k*_loss_ and high values of *V*_D_, consistent with low turnover and high storage capacity for dopamine.

When the rate constant for the loss of metabolites to cerebrospinal fluid (CSF) (*k*_loss_) is very small, long dynamic PET sessions may be necessary for the accurate quantitation of the rate of slow continuous loss of metabolites from brain tissue. For this reason, we extended the recording to three hours for these young normal control subjects with different sensation-seeking levels, in order to better differentiate subtle changes in the low magnitude range of *k*_loss_ estimates. As predicted, the individual kinetic estimates of the group had a very limited range, which replicated the results of earlier studies of FDOPA with the inlet-outlet model (IOM) (Kumakura & Cumming, 2009; Vernaleken et al., 2008).

The correlation of ZSSS scores with parametric maps of *V*_D_ estimates revealed a cluster of voxels in the left dorsal striatum, where the estimates of *k*_loss_ correlated negatively, and the estimates of *V*_D_ correlated positively, with the ZSSS scores. We attribute the correlations both to a decreased effect of the action of monoamine oxidase (MAO) activity in association with increased trapping of radiolabeled fluoro dopamine and its metabolites in the dopaminergic terminals of the dorsal striatum of the highly sensation-seeking individuals, linked to increased reuptake of dopamine that allows efficient re-trapping of previously released DA. Due to the structure of the analysis, the three kinetic estimates are interrelated, such that *k*_loss_ and *V*_D_ maintain a hyperbolic relationship. The metabolism of intracellular fluorodopamine is mainly subject to degradation by MAO, but the velocity of the entire metabolism, including the breakdown by catechol-O-methyltransferase (COMT) and passive efflux to CSF, is reflected in the magnitude of *k*_loss_ (Cumming et al., 1995; Matsubara et al., 2011). Lower rates of fluorodopamine elimination (i.e., lower values of *k*_loss_) imply increased vesicular sequestration of fluorodopamine from the cytosol, associated with increased synaptic release and redistribution to the extracellular space. Thus, reduced values of *k*_loss_ naturally lead to increased vesicular fluorodopamine trapping, consistent with the results of increased *V*_D_ in the highly sensation-seeking individuals.

The intracellular dopamine concentration is regulated by a complex mechanism involving DOPA decarboxylase, vesicular transporters (VMAT2), dopamine reuptake sites (DAT), and monoamine oxidase (MAO) that cannot readily be disentangled by current methods. Instead, we introduce a sequestration index (SI), defined as the ratio of vesicular to non-vesicular dopamine contents, indicative of the degree of sequestration, or protection of fluorodopamine from degradation. The magnitude of the SI can be evaluated from the magnitudes of *k*_loss_, and the reported turnover constant of MAO (*k*_MAO_) (Gjedde et al., 2010) (table 2.4, page 93) of 20 min^− 1^, combined into the expression (*k*_MAO_/*k*_loss_)^− 1^. The value of SI for the dorsal striatum cluster is approximately 6,000, in good agreement with the ratio obtained from the values of the vesicular (48 nmol cm^− 3^) and non-vesicular (0.015 nmol cm^− 3^) dopamine contents (Gjedde et al., 2010) (table 2.4, page 93). Ignoring extracellular dopamine contents that are orders of magnitude lower than the size of dopamine storage in vesicles, the effective distribution volume (*V*_ED_) can be simplified to,

1 $$ V_{\text{ED}} = V_i (1 + SI)  $$

where *V*_*i*_ is the distribution volume of fluorodopamine in the cytosolic in non-vesicular space. Adding the effective plasma (*V*_0_) and the precursor pool (*V*_*f*_) volumes to both sides yields the expression,

2 $$ V_{\text{D}} = [V_i(1+ SI)] + V_f + V_0  $$

whereof we know that the magnitude of *V*_*f*_ does not covary with the ZSSS scores in the present study, because of the unchanged dopamine synthesis capacity. Based on the result of the linear correlation between values of *V*_D_ and the ZSSS scores, we conclude that sensation-seeking propensity scored as ZSSS in the healthy volunteers is proportional to the product of *V*_*i*_ and SI,

3 $$ ZSSS = \alpha V_i SI + \beta  $$

where *α* and β are constants. In the relationship, *V*_*i*_ represents the non-vesicular concentration of fluorodopamine, relative to arterial FDOPA. This means that sensation-seeking propensity is inversely associated with dopamine degradation activity, via regulation of the non-vesicular DA concentration.

MAO modulates human mood, animals’ behavior and vulnerability to psychiatric disorder. MAO-B activity in blood platelets has been used as a surrogate marker of central MAO-B activity (Bench et al., 1991; Chen et al., 1993; Zuckerman et al., 1980) and sensation-seeking has been found to be inversely proportional to platelet MAO-B activity (Fowler et al., 1980) which supports the positive correlation between the magnitudes of *k*_loss_ and measures of platelet MAO-B activity, as presented in Fig. 4c and d.

**Fig. 5:**
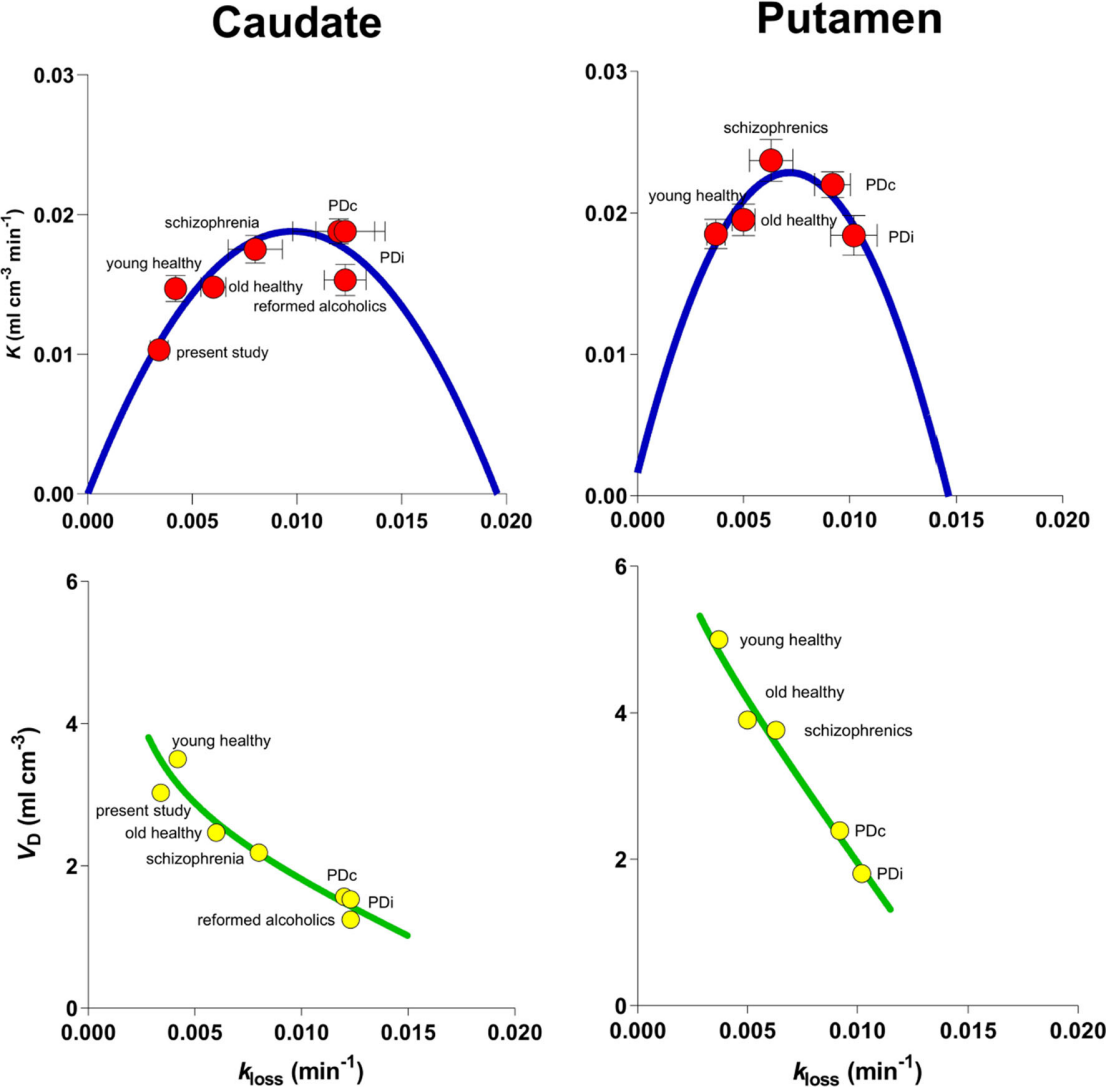
State or trait differences among patient and control populations, reported by Kumakura et al. (2009) for caudate nucleus and putamen, (Kumakura & Cumming, 2009) indicating average variables associated with different conditions and traits involving the relationship among the variable estimates derived from analysis of FDOPA transfer and metabolism in human brain, as well as the average relations among *K*, *V*_D_ and *k*_loss_ in caudate nucleus obtained in the present study where we detected no significant cluster in putamen and did not estimate values of *K*, *V*_D_ and *k*_loss_ for this region

The region of the left dorsal striatum identified in the present study partially overlaps with another cluster of voxels (Supplementary Figure 7) that was reported to show significantly elevated dopamine turnover in abstinent alcoholic patients (Kumakura et al., 2013). However, the mean *k*_loss_ magnitude of the high sensation-seekers was smaller by at least 70% of the value reported for alcoholic patients (0.0123 min^− 1^), while the FDOPA transfer coefficient or clearance (*K*), described as a measure of synthesis capacity, remained unchanged in reference to the *K* vs *k*_loss_ correlation previously presented for several diseases (Fig. 5; modified from Kumakura & Cumming, 2009). The decline of the magnitude of *k*_loss_ with increasing sensation-seeking propensity may be associated with transient positive mood or disinhibited impulsivity within the original healthy homeostasis. The increased *K* with reversed *k*_loss_ rise that we reported for abstinent alcoholics may reflect fixed negative mood after long-term hysteresis of repeated alcohol effects, as discussed in the spiraling addiction cycle model (Koob & Le Moal, 2001).

The hypothesis predicted a positive relation between the synthesis capacity and clearance-dependent turnover rate, as implied by studies of schizophrenia, where the condition is held to reflect a specific neurobiological trait rather than a variable state of mind. Thus, the hypothesis cannot be upheld in its original form. However, while schizophrenia can be considered a trait, in which the regulation of dopamine synthesis is constitutionally abnormal, it now appears that sensation-seeking may reflect a state of mind, in which the dopamine loss-dependent turnover varies in inverse proportion to the actual score of sensation-seeking as a form of addictive learning. The inverse proportionality raises the interesting possibility of a state in which dopamine reuptake is also reduced, in the same manner as that of methylphenidate blockade of dopamine reuptake, leading to decrease of *k*_loss_ (Schabram et al., 2014). In such a state, chronically elevated vesicular and extracellular dopamine concentrations accompany chronically elevated dopamine receptor numbers in the striatum, with ventral predominance (Gjedde et al., 2010).

Findings suggestive of a state of mind underlying sensation-seeking propensity are summarized in Fig. 5, where the average current results are shown together with group averages reported in the literature (Kumakura & Cumming, 2009; Kumakura et al., 2013). The figures, presenting the individual (Fig. 4) and population (Fig. 5) differences, respectively, suggest that a state of sensation-seeking is modulated by the fate of presynaptic dopamine, as influenced by the action of MAO (Fig. 4a, c, and d), together with the extracellular dopamine concentration and receptor density (Fig. 4e, f, g, and h).

In contrast, the traits shown in Fig. 5 define an inverted-U shape of the transfer coefficient (*K*), which appears to delineate a basic dopaminergic function that is insensitive to the actual state of sensation-seeking (Fig. 4f). The population averages imply constitutional differences that are independent of differences of sensation-seeking propensity among the young volunteers studied here, but which may with time arise from the continued maintenance of a specific state, such as the one associated with sensation-seeking activities in the young volunteers. Trait and state fluctuations can be said to interact as results of an interplay between acute and chronic conditions. A trait of sensation-seeking has long been held to be a stable personality construct.

However, during adolescence, the majority of sensation-seeking propensities can change rapidly over time (Lynne-Landsman et al., 2011). A single dose of the D_2/3_ agonist cabergoline directly affected human risk-taking, and the effect depended on baseline differences of the sensation-seeking propensity (Norbury et al., 2013).

Previously, traits have been described as density distributions of states, in which the traits depend on clusters of the states of mind (Fleeson, 2001). Importantly, neuroimaging studies show that states of mind modulate neuronal activity of striatal structures (Beauregard, 2007). Indeed, effects of a psychostimulant (Kaasinen et al., 2004) and of a therapeutic drug for Parkinson’s disease (De la Fuente-Fernández et al., 2001) both led to reduction of raclopride binding, suggesting that expectations play a pivotal role in the modulation of dopaminergic neurotransmission. The distinction between state and trait is important to the interpretation of the present findings. In essence it is a question either of learning from sensation seeking, followed by changes of dopaminergic neurotransmission, or of permanent properties of the dopaminergic neurotransmission that compel the individuals to seek sensations.

Several studies reveal a relationship among the variables of FDOPA accumulation in the striatum of humans, in whom the volume of distribution varies inversely with the elimination rate, as shown in Figs. 3 and 5. This relationship implies that the increased turnover of dopamine that underlies the learning of behavior may be the fundamental factor in the mechanism that serves to store dopamine in vesicles and hence to make the learning addictive. The present results confirm that metabolic activity, in step with the vesicular dopamine storage and sequestration in the left dorsal striatum, plays an important role in the learning of behavioral phenotypes of sensation-seeking.

### Limitations

The representative transaxial brain maps of the magnitude of quadratic coefficients were calculated voxelwise by least-squares optimization of the linear relationship. Here, the Zuckermann sensation-seeking score is the explanatory variable and the parameters of the inlet-outlet model of each voxel are the dependent variables that we calculated without correction for multiple comparisons, having decided that the preselection of regions-of-interest rendered the determination of multiple comparisons of little benefit.

## Conclusion

Sensation-seeking behavior is held to be linked to premorbid personality characteristics in patients with addictive disorders. We used PET and the labeled DA precursor FDOPA to investigate the relationship between sensation-seeking and dopamine turnover in healthy male volunteers. FDOPA undergoes decarboxylation to fluorodopamine, followed by metabolism and elimination. Quantitative image analysis revealed that highly sensation-seeking individuals have lower fluorodopamine turnover (higher fluorodopamine retention) in left dorsal striatum, where dopamine signaling serves to control motivated behavior. The average turnover rate for loss of DA metabolites from brain tissue correlated negatively with the ZSSS scores, whereas the apparent volume of distribution of fluorodopamine correlated positively. We suggest that the elevated DA store of high sensation-seekers reveals a state of mind which is the cause rather than the consequence of a neurobiological trait.

## Electronic supplementary material

Below is the link to the electronic supplementary material.

(PNG 162 KB)

(PNG 106 KB)

## Data Availability

The data sets used and/or analyzed during the current study are available from the corresponding authors on reasonable request.
