# Supplementary figures and images for: On the learning of addictive behavior: Sensation-seeking propensity predicts dopamine turnover in dorsal striatum
Source: Brain Imaging Behav. 2021 Aug 21;16(1):355–65. doi: 10.1007/s11682-021-00509-5 (PMC8825434; doi:10.1007/s11682-021-00509-5)

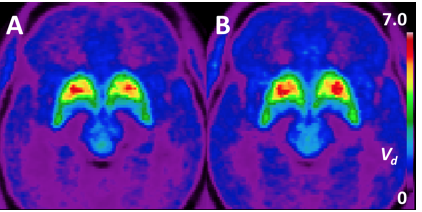

Supplement: Supplementary file 1 — (PNG 162 KB) [file 11682_2021_509_MOESM1_ESM.png]

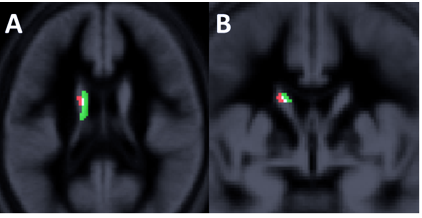

Supplement: Supplementary file 2 — (PNG 106 KB) [file 11682_2021_509_MOESM2_ESM.png]
